# Interaction of Sulforaphane with DNA and RNA

**DOI:** 10.1371/journal.pone.0127541

**Published:** 2015-06-01

**Authors:** Farzaneh Abassi Joozdani, Faramarz Yari, Parvaneh Abassi Joozdani, Shohreh Nafisi

**Affiliations:** 1 Department of Biology, IAU, Science and Research Branch, Tehran, Iran; 2 Department of Chemistry, IAU, Central Tehran Branch, Tehran, Iran; 3 Department of Dermatology, University of California, San Francisco, California, United States of America; The University of North Carolina at Charlotte, UNITED STATES

## Abstract

Sulforaphane (SFN) is an isothiocyanate found in cruciferous vegetables with anti-inflammatory, anti-oxidant and anti-cancer activities. However, the antioxidant and anticancer mechanism of sulforaphane is not well understood. In the present research, we reported binding modes, binding constants and stability of SFN–DNA and -RNA complexes by Fourier transform infrared (FTIR) and UV–Visible spectroscopic methods. Spectroscopic evidence showed DNA intercalation with some degree of groove binding. SFN binds minor and major grooves of DNA and backbone phosphate (PO_2_), while RNA binding is through G, U, A bases with some degree of SFN–phosphate (PO_2_) interaction. Overall binding constants were estimated to be K_(SFN–DNA)_=3.01 (± 0.035)×10^4^ M^-1^ and K_(SFN–RNA)_= 6.63 (±0.042)×10^3^ M^-1^. At high SFN concentration (SFN/RNA = 1/1), DNA conformation changed from B to A occurred, while RNA remained in A-family structure.

## Introduction

**Fig 1:**
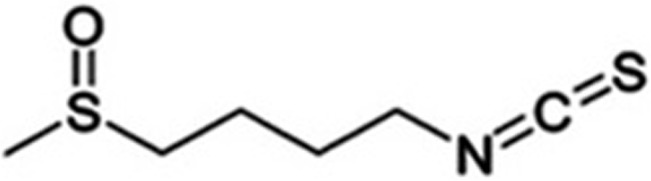
Chemical structure of sulforaphane (SFN).

Sulforaphane (1-isothiocyanato-4-(methyl-sulfinyl)) butane, a molecule within the isothiocyanate group of organosulfur compounds (Fig 1); is the most characterized isothiocyanate found at high levels in cruciferous vegetables such as broccoli, cabbages, kale, Brussels sprouts, radish, and mustard [1]. It has shown anti-inflammatory, antibiotic, antioxidant and anticarcinogenic properties [2–10]. Sulforaphane chemoprevention properties against cancer are through both “blocking” and “suppressing” effects [11]. Blocking function is inhibiting Phase 1 metabolism enzymes which can activate procarcinogenic compounds to their carcinogenic metabolites and induce phase 2 metabolic enzymes. Suppressing effects revealed modulating diverse cellular activities and inhibiting growth of transformed cells [11, 12]. SFN acts as an antioxidant by increasing reduced glutathione levels as well as inducing cell cycle arrest and apoptosis [13, 14] by regulation of many molecules including Bcl-2 family proteins, caspases, p21, cyclins and cdks [12, 13]. Gene expression of phase 2 proteins is regulated by three cellular components; Kelch-like ECH-associated protein 1 (Keap 1); Nuclear factor (eryhthroid-derived 2)-like 2 (Nrf2); and ARE (anti-oxidant response element). Under normal conditions, Nrf2 is sequestered in cytoplasm by Keap 1 and is subject to ubiquitination and proteasomal degradation. In the presence of SFN, it targets and chemically modifies specific and highly reactive cysteine thiols of Keap 1 resulting conformational changes and dissociation of Nrf2 from Keap 1, and stabilization of Nrf2. Nrf2 undergoes nuclear translocation and binds to ARE and activates transcription of phase 2 genes [15– 21].

Even though much is reported about antitumor activities of sulforaphane, there has been no report on the molecular aspects of sulforaphane interaction with DNA and RNA in aqueous solution. RNA, a versatile molecule playing essential roles in many biological processes is an attractive target for potential therapeutics. Recent progress in antiviral research has been mainly based on targeting RNA molecules for therapeutic intervention. Development of molecules capable of controlling RNA activity is now a focus of medicinal and chemico-biological research [22–24]. Understanding the mechanism of sulforaphane action may expedite development of new drugs based on SFN. So far, little is known about sulforaphane interaction with individual DNA and RNA. Thus, we compared SFN interaction with DNA and RNA in aqueous solution at pH 6.5–7.5 with sulforaphane / DNA, RNA (P) molar ratios of 1/80 to1/1 by FTIR and UV measurements. Structural analyses regarding drug binding site, binding constant, and DNA, RNA secondary structures are presented here. Our spectroscopic results provided structural analysis of sulforaphane—biopolymer interactions.

## Materials and Methods

### Materials

DNA sodium salt, and Baker’s yeast RNA sodium sulforaphane were purchased from Sigma Chemical (St. Louis, MO) and used without further purification. To check the protein content of DNA and RNA solutions, the absorbance bands at 258 and 280 nm were used. The A_258_/A_280_ ratio was 1.80 for DNA and A_260_/A_280_ ratio 2.10 for RNA, showing that DNA and RNA samples were sufficiently free from protein [25]. Other chemicals were of reagent grade and used without further purification.

#### Preparation of stock solutions

Sodium–DNA or sodium–RNA were dissolved to 0.5% w/v (0.0125M (phosphate)) in NaCl (0.1 M) solution for 24 h with occasional stirring to ensure the formation of a homogeneous solution. The final concentration of the calf thymus DNA and yeast RNA solution were determined spectrophotometrically at 258 nm using molar extinction coefficient ε_259_ = 6600 cm^-1^ M^-1^ (DNA) and ε_258_ = 9250 cm^-1^ M^-1^ (RNA) (expressed as molarity of the phosphate groups) [26]. The definite amounts of sulforaphane (0.05–12.5 mM) were dissolved in water and added dropwise to biopolymers solutions (12.5mM) to attain the desired drug/DNA and RNA (P) molar ratios (r) of 1/80, 1/40, 1/20, 1/10, 1/5 and 1/1 with a final DNA (P) and RNA concentrations of 6.25 mM. At higher concentrations of sulforaphane /RNA (r = 1/1), the experiments could not be continued due to DNA gel formation. The pH values of the solutions were adjusted at 7.0±0.2 using NaOH solution. The infrared spectra were recorded 1h after mixing of the drugs with DNA or RNA solution. For UV measurements, the drug concentrations of 5×10^–6^–1×10^–4^ M were used with constant DNA or RNA concentration of 5×10^–4^ M.

### FTIR spectroscopy measurements

Infrared spectra were recorded on a Nicolet FTIR spectrometer (Magna 550) equipped with a liquid-nitrogen-cooled HgCdTe (MCT) detector. The spectra of drugs/DNA or drug/RNA solutions were taken using a cell assembled with ZnSe windows. Spectra were collected and treated using the OMNIC software supplied by the manufacturer of the spectrophotometer. The spectra of the solutions were recorded after 1h incubation of drugs with DNA or RNA solutions. The bands were measured in triplicates (three individual samples of the same DNA or RNA and drug concentrations). For each spectrum, 100 scans were collected with resolution of 4 cm^-1^. The difference spectra [(polynucleotide solution+ drug solution)-(polynucleotide solution)] were obtained using a sharp DNA and RNA band at 968 cm^-1^ as internal reference [27,28]. This band, which is due to sugar C-C and C-O stretching vibrations, exhibits no spectral change (shifting or intensity variation) upon drug–DNA,—RNA complexation, and is cancelled out upon spectral subtraction.

**Fig 2:**
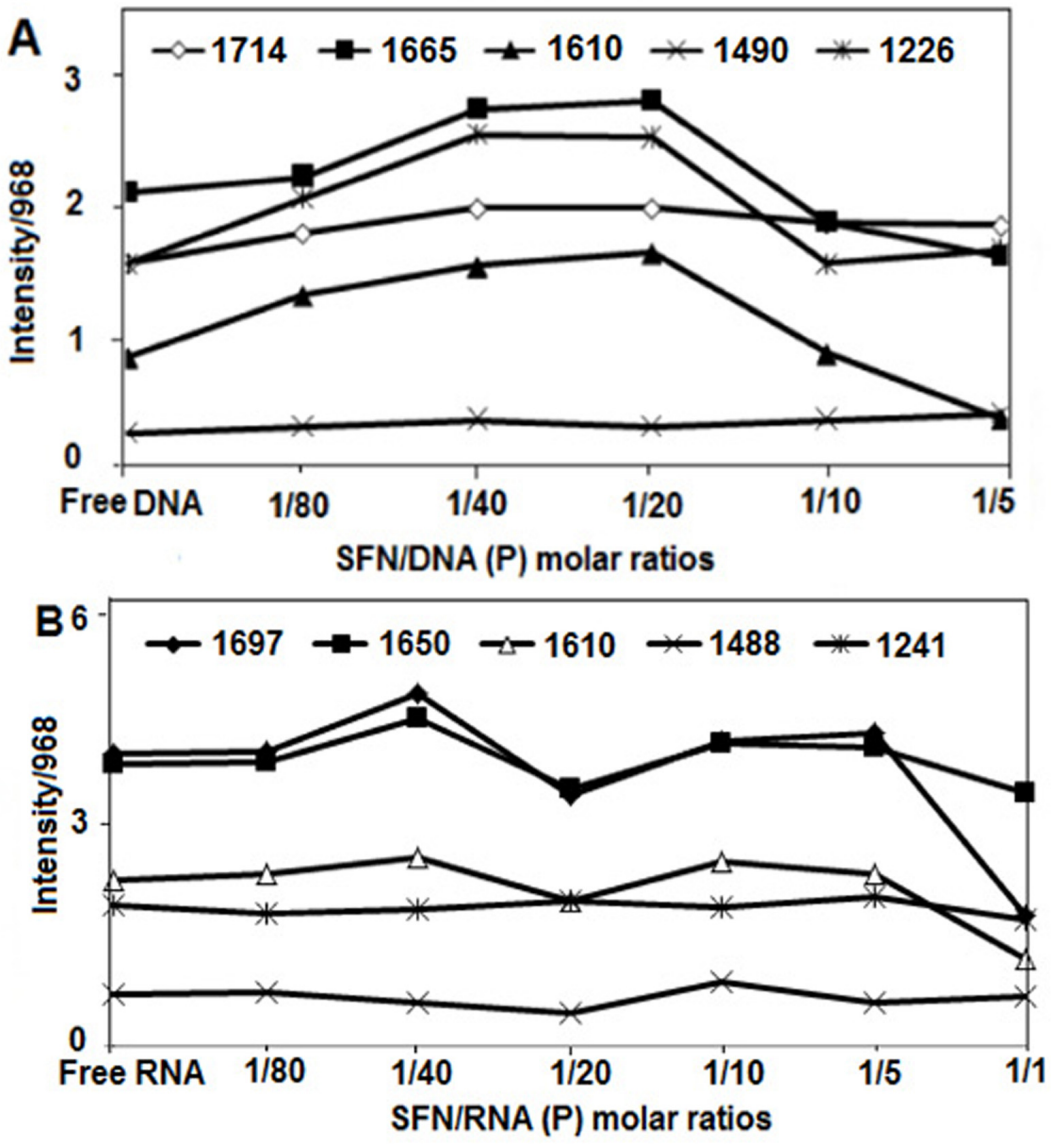
Intensity ratio variations for several DNA and RNA in-plane vibrations as a function of SFN concentration. (A) Intensity ratios for DNA bands at 1714 (G, T), 1665 (T, G, A, C), 1610 (A), 1490 (C,G) and 1226 (PO2 asymmetric) referenced to the DNA band at 966 cm^-1^. (B) Intensity ratios for the RNA bands at 1697 (G, U), 1650 (U, G, A, C), 1610 (A), 1488 (C,G) and 1241 (PO2 asymmetric) referenced to the RNA band at 969 cm^-1^.

The intensity ratios of the bands due to several DNA in-plane vibrations related to A-T and G-C base pairs and RNA in-plane vibrations related to A-U and G-C base pairs and the phosphate stretching vibrations were measured with respect to the reference bands at 968 cm^-1^ (DNA and RNA) as a function of sulforaphane concentrations with an error of ±3%. Similar intensity variations have been used to determine the ligand binding to DNA and RNA bases and backbone phosphate groups [29]. The plots of the relative intensity (R) of several peaks of DNA in-plane vibrations related to A-T and G-C base pairs and the phosphate stretching vibrations such as 1714 (guanine), 1665 (thymine), 1610 (adenine), 1490 (cytosine), 1226 (asymmetric PO_2_), and 1088 cm^-1^ (symmetric PO_2_) and RNA in-plane vibrations related to A-U and G-C base pairs and the phosphate stretching vibrations such as 1697 (guanine), 1650 (uracil), 1610 (adenine), 1488 (cytosine), and 1241cm^-1^ (phosphate groups) versus sulforaphane concentrations were obtained after peak normalization using R_i_ = I_i_/I_968_, where I_i_ is the intensity of the absorption peak for pure DNA or RNA in the complex with i as ligand concentration, and I_968_ is the intensity of the 968 cm^-1^ peak (DNA and RNA internal reference) (Fig 2A and 2B).

### Absorption spectroscopy

The absorption spectra were recorded on a LKB model T90 + UV/Vis Spectrometer PG Instruments ltd, quartz cuvettes of 1cm were used and the absorption spectra recorded with drug concentrations of 5×10^–6–^1×10^–4^ M and constant polynucleotide concentration of 5×10^–4^ M.

The binding constants of the drug-DNA or drug-RNA complexes were calculated as reported [30]. It is assumed that the interaction between the drug [L] and the substrate [S] is 1:1; for this reason a single complex SL (1:1) is formed. The relationship between the observed absorbance change per centimeter and the system variables and parameters is as follow;

$$\frac{\Delta \text{A}}{b}=\frac{S_{t}K_{11}\Delta \varepsilon_{11}\left[L\right]}{1+K_{11}\left[L\right]}$$ (1)

where ΔA = A—A_0_ from the mass balance expression S_t_ = [S] + [SL], we get

[S] = S_t_/(1 + K_11_[L]). Eq (1) is the binding isotherm, which shows the hyperbolic

dependence on free ligand concentration. The double-reciprocal form of plotting the rectangular hyperbola $\frac{1}{y}=\frac{f}{d}\cdot \frac{1}{x}+\frac{e}{d}$, is based on the linearization of Eq (1) according to the following equation,

$$\frac{b}{\Delta \text{A}}=\frac{1}{S_{t}K_{11}\Delta \varepsilon_{11}\left[L\right]}+\frac{1}{S_{t}\Delta \varepsilon_{11}}$$ (2)

Thus the double reciprocal plot of 1/ΔA versus 1/[L] is linear and the binding constant can be estimated from the following equation:

$$K_{11}=\frac{intercept}{slope}$$ (3)

### Molecular modeling and docking

The crystal structures of two DNA–drug and RNA-drug complexes were selected from Protein Data Bank (Web address: http://www.rcsb.org) PDB ID: 1BNA-DNA and PDB ID: 2R22 and PDB ID:3CZW [31]. The crystal structure of the synthetic DNA dodecamer d(CpGpCpGpApApTpTpCpGpCpG) has been used for simulation. Sulforaphane docked onto the DNA and RNA from the crystal structures. For every individual model, the correlation between calculated binding and experimental values were analyzed to determine the most representative model. To determine the preferred the binding sites on DNA, docking studies were performed by AutoDock 4.0.1 software ([32]; Web address: http://autodock.scripps.edu). The sulforaphane structure was extracted from Pubchem (CID 16213697,10114) file. In order to use the structures for docking, the universal force field (for drugs) [33] and Merck molecular force field 94 (for macromolecule) [34] were short minimized. Docking to macromolecule was carried out using the Lamarckian genetics algorithm. For the local search, the so-called pseudo-Solis and Wets algorithm was applied using a maximum of 300 iterations per local search [35]. In AutoDock, the overall docking energy of a given drug molecule in its active site is expressed as follows:

$$\Delta \text{G}=\Delta {\text{G}}_{\text{vdW}}\sum_{\text{i},\text{j}}\left(\frac{{\text{A}}_{\text{ij}}}{{\text{r}}_{\text{ij}}^{12}}-\frac{{\text{B}}_{\text{ij}}}{{\text{r}}_{\text{ij}}^{6}}\right)+\Delta {\text{G}}_{\text{hbond}}\sum_{\text{i},\text{j}}\left(\frac{{\text{C}}_{\text{ij}}}{{\text{r}}_{\text{ij}}^{12}}-\frac{{\text{D}}_{\text{ij}}}{{\text{r}}_{\text{ij}}^{10}}+{\text{E}}_{\text{hbond}}\right)+\Delta {\text{G}}_{\text{elec}}\sum_{\text{i},\text{j}}\frac{{\text{q}}_{\text{i}}-{\text{q}}_{\text{j}}}{\varepsilon \left({\text{r}}_{\text{ij}}\right){\text{r}}_{\text{ij}}}+\Delta {\text{G}}_{\text{tor}}{\text{N}}_{\text{tor}}+\Delta {\text{G}}_{\text{sol}}\sum_{{\text{i}}_{\text{C}},\text{j}}{\text{S}}_{\text{i}}{\text{V}}_{\text{j}}{\text{e}}^{\left(-{\text{r}}_{\text{ij}}^{2}/2\sigma^{2}\right)}$$ (4)

In Eq (4), ΔG _vdW_, ΔG _hbond_, ΔG _elec_, ΔG _tor_, and ΔG _sol_ are free energy coefficients of van der Waals, hydrogen bond, electrostatic interactions, torsional term, and desolvation energy of oligonucleotide–drug complex, respectively. r_ij_, A_ij_, B_ij_, C_ij_, and D_ij_ represent the interatomic distance, the depths of energy well, and the equilibrium separations between the two atoms, respectively. The first three terms are in vacuo force field energies for intermolecular interactions. The fourth term accounts for the internal steric energy of the drug molecule. The energies used and reported by AutoDock should be distinguished: there are docked energies, which include the intermolecular and intramolecular interaction energies, and are used during dockings, and predicted free energies, which include the intermolecular energy and the torsional free energy, and are only reported at the end of a docking [36, 37]. We converted between the binding constant, K_binding_ and the binding free energy change of binding, ΔG_binding_, using the following equation:

$$\Delta G_{binding}=-RT\;\text{ln}\;K_{binding}$$ (5)

where R is the gas constant, 1.987 cal K^-1^ mol^-1^, and T is the absolute temperature, assumed to be room temperature, 298.15 K.

In order to analyze and display docking results, we used AutoDock Tools 1.5.4 (ADT) ([38]; Web address: http://mgltools.scripps.edu) and UCSF Chimera 1.6.1([39];Web address: http://www.cgl.ucsf.edu/chimera).

## Results and Discussion

### Infrared spectra of sulforaphane–DNA complexes

**Fig 3:**
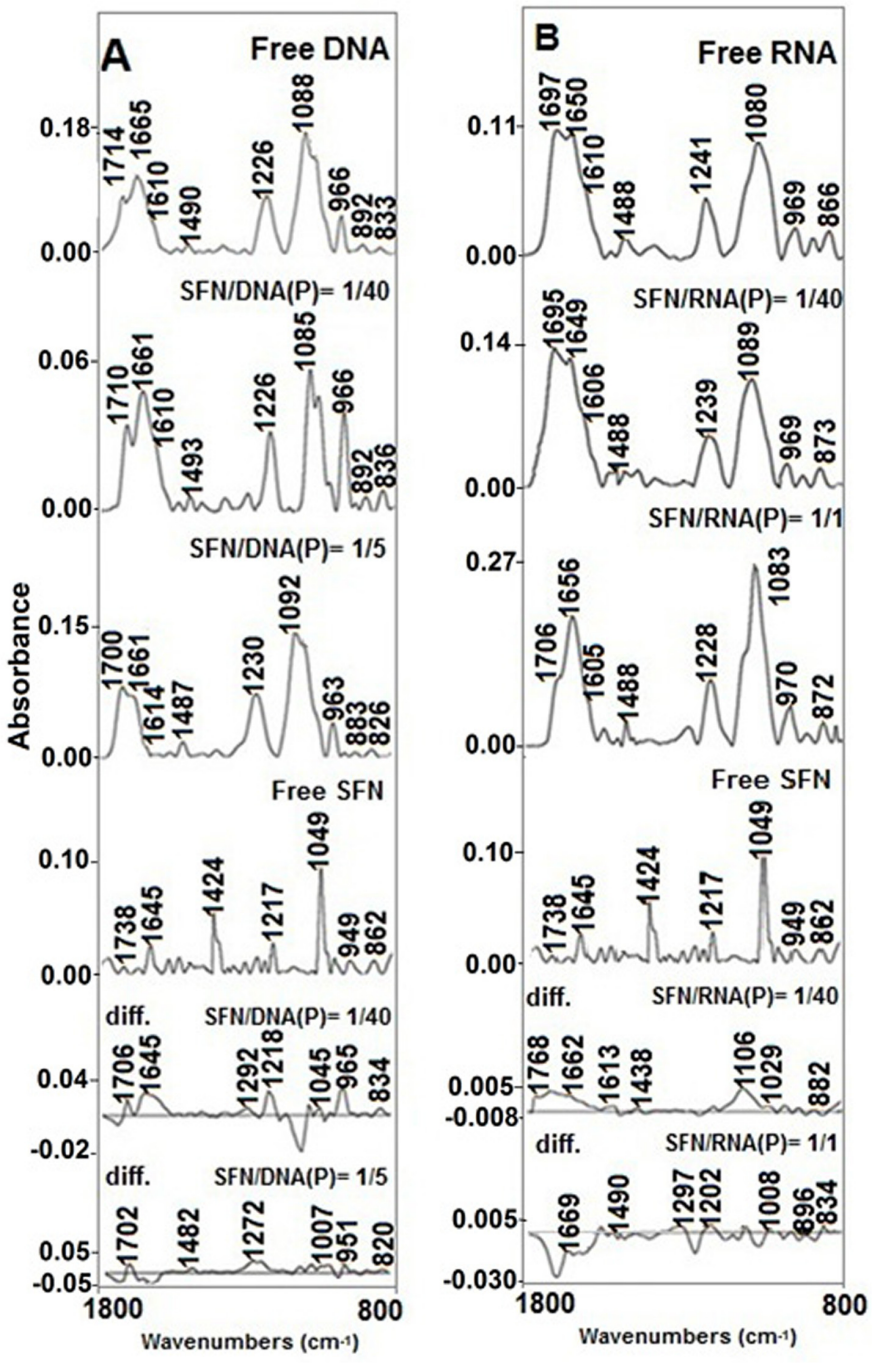
FTIR spectra in the region of 1800–800 cm^-1^ for sulforaphane (SFN), calf thymus DNA (A) and yeast RNA adducts (B) in aqueous solution at pH = 7. DNA or RNA and two complexes spectra were obtained at various SFN/DNA and-RNA (phosphate) molar ratios (three two spectra); sulforaphane and two difference spectra (bottom three spectra).

Evidence of sulforaphane–DNA complexation comes from the infrared spectroscopic results shown in Figs 3A and 2A. The spectral changes (intensity and shifting) of several prominent DNA in-plane vibrations at 1714 (G,T; mainly G), 1665 (T, G, A, and C; mainly T), 1610 (A, C; mainly A), 1490 (C, G; mainly C), 1226 (PO_2_ asymmetric stretch) and 1088 cm^-1^ (PO_2_ symmetric stretch) [40–42] were monitored at various SFN concentrations binding to DNA (Fig 3A).

Major interaction was observed with the bases, mainly with minor groove; A-T rich region and guanine of DNA duplex as evidenced by FTIR spectra in Figs 3A and 2A. At low SFN concentrations (r = 1/80 to 1/20), the intensity of the thymine, guanine and adenine bands increased which can be ascribed to the interaction of SFN with bases. At r = 1/10, major decrease in intensity of the bases mainly thymine and adenine and to a lesser extent with guanine can be related to DNA destabilization upon SFN interaction. The observed intensity changes were accompanied by shifting of the guanine band at 1714 to 1710 (r = 1/40) and 1700 cm^-1^ (r = 1/5), thymine band at 1665 to 1661 cm^-1^ (r = 1/40, 1/5), adenine band at 1610 to 1614 cm^-1^ (r = 1/5) (Fig 3A). Major spectral changes (intensity and shifting) of the DNA bases upon sulforaphane interaction is indicative of drastic participation of adenine, thymine and guanine bases in SFN complexation. No major shifting was observed for the cytosine which demonstrated no major participation of cytosine in SFN interaction.

At low SFN concentrations (r = 1/40), no major intensity changes was observed for the phosphate band at 1226 cm^-1^, however at high concentrations (r = 1/5), it shifted to 1230 cm^-1^ which can be related to phosphate interaction with SFN at higher concentrations (Fig 3A). In addition to major spectral shifting of the PO_2_ asymmetric band, the relative intensities of the asymmetric (*v*_as_) and symmetric (*v*_s_) vibrations were altered upon phosphate interaction [40]. The *v*_s_ PO_2_ (1088 cm^-1^) and *v*_as_ PO_2_ (1226 cm^-1^) were changed, with the ratio *v*_*s*_ */v*_*as*_ going from 2.16 (free DNA) to 1.95 (SFN–DNA complexes) in various molar ratios of sulforaphane–DNA.

In the difference spectra of sulforaphane-DNA (r = 1/40), the positive features at 1706, 1645, and 1218 cm^-1^ (Fig 3B, Diff. r = 1/40) are due to the intensity increase of the guanine, thymine, and phosphate bands upon sulforaphane complexation and approves major interaction of sulforaphane with guanine N7, thymine O2 and backbone PO_2_ group.

**Fig 4:**
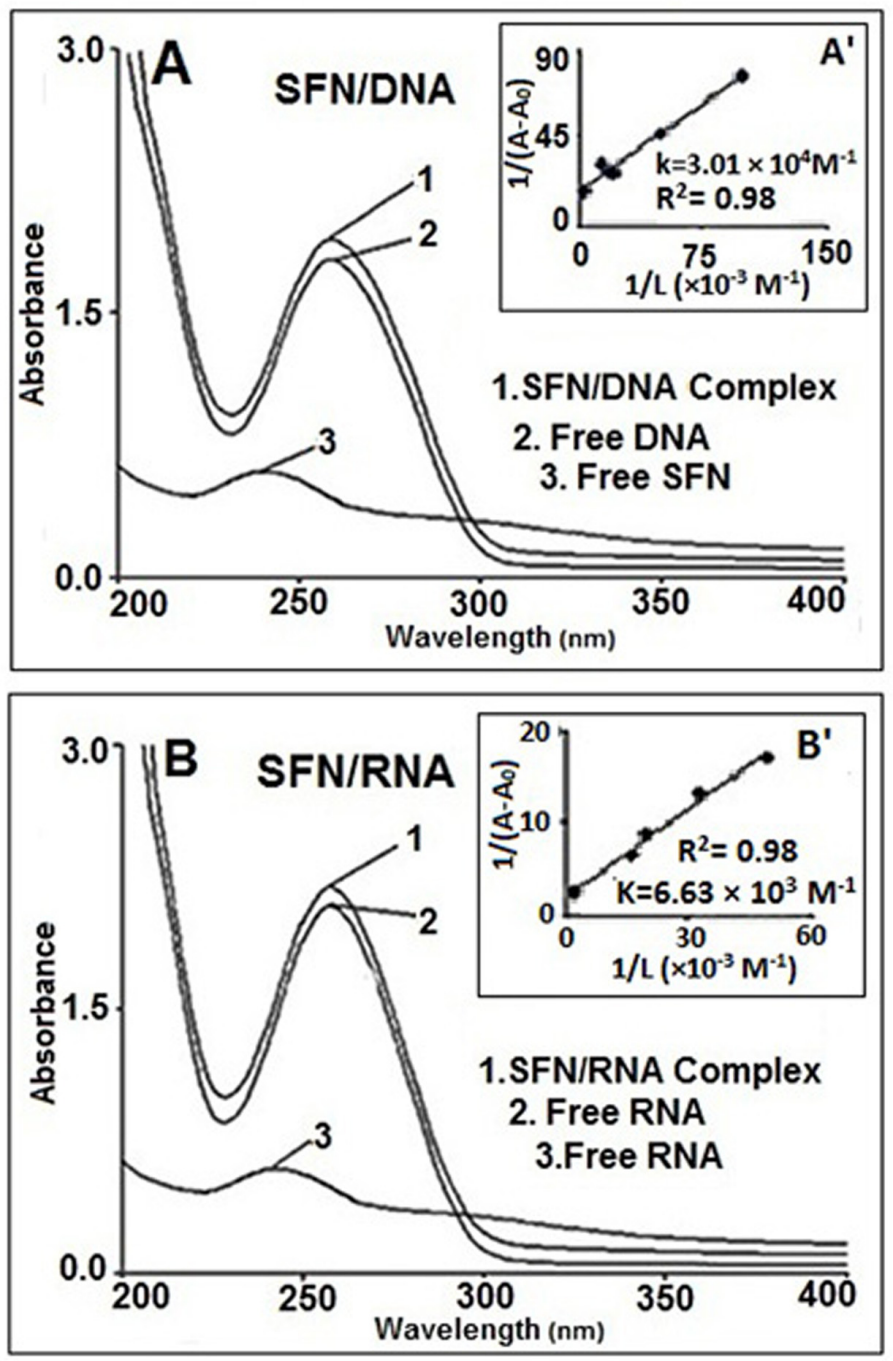
Ultraviolet–visible results of SFN and calf thymus DNA and its Complexes (A), and yeast RNA and its complex (B): (1) SFN–DNA and SFN–RNA complexes; (2) free DNA or free RNA (0.5 mM); (3) free SFN (0.5 mM). Plot of 1/(A-A_0_) versus (1/drug concentration) for SFN and calf thymus DNA complexes (A^'^), and plot of 1/(A-A_0_) versus (1/drug concentration) for SFN and yeast RNA complexes (B^'^), where A_0_ is the initial absorbance of DNA (259 nm) or RNA at (258 nm) and A is the recorded absorbance at different SFN concentrations (5×10^–6–^1×10^–4^ M) with constant DNA or RNA concentrations of 0.5 mM at pH 7.

The UV results indicated intensity increase in the DNA band at 259 nm upon SFN interaction (Fig 4A). Similar spectral changes were observed in other DNA adducts [43]. On the other hand, the UV band at 245 nm characteristic of sulforaphane absorption showed major red shift and appeared at 259 nm upon DNA interaction. The observed major shifting of sulforaphane band is indicative of structural alterations of the SFN upon DNA complexation (Fig 4A).

### Infrared spectra of sulforaphane–RNA complexes

Evidence related to sulforaphane-RNA complexation comes from the infrared spectroscopic results shown in Figs 3B and 2B. In the sulforaphane–RNA complexes, SFN binds mainly to guanine and uracil bases and to a lesser extent to adenine bases, as well as the backbone PO_2_ group (Fig 3B). Evidence for this comes from spectral changes of the bases and phosphate bands upon SFN interaction (Figs 3B and 2B).

No major spectral changes (shifting and intensity) were observed for the bases and phosphate bands at low concentration (r = 1/80). However at r = 1/40, increase in the intensity of guanine, uracil and to a lesser extent adenine bands can be related to sulforaphane interaction with these bases. At r = 1/20, reduction in intensity of the G, U and A bands arise from RNA stabilization upon SFN interaction (Fig 2B). No major interaction was observed for adenine, guanine and uracil bands at higher concentrations (r = 1/10, 1/5) due to minor intensity changes of the bands upon SFN interaction (Fig 2B). At r = 1/1, RNA aggregation occurred upon SFN interaction due to major decrease in intensity of guanine, uracil and adenine bands.

The intensity changes were accompanied by major shifting of the bases bands especially at higher SFN concentrations (r = 1/5); the guanine band at 1697 shifted to 1706 cm^-1^, the uracil band at 1650 shifted to 1656 cm^-1^, the adenine band at 1610 shifted to 1605 cm^-1^. The major spectral changes of RNA bands upon sulforaphane interaction can be related to major interaction of SFN with RNA.

For the backbone PO_2_ asymmetric stretching, no major shifting was observed at low SFN concentration; 1241 to 1239 cm^-1^ (r = 1/40), however, a drastic shifting was observed at higher concentration; 1241 to 1228 cm^-1^ (r = 1/1) (Fig 2A). Some intensity changes in the backbone phosphate group were also observed upon sulforaphane interaction (r = 1/40 to 1/1). The shifting and intensity changes in the phosphate band upon sulforaphane interaction can be related to the interaction of SFN with phosphate backbone group especially at higher concentrations (Fig 2B).

It is worth mentioning that minor spectral changes for cytosine band at 1488 cm^-1^ upon sulforaphane interaction with RNA is indicative of minor participation of cytosine in SFN complexation (Figs 3B and 2B). Similar spectral changes were observed in other RNA adducts [44].

In the difference spectra of sulforaphane-RNA (r = 1/40), the positive features at 1768, 1662, and 1106 cm^-1^ (Fig 3B, Diff. r = 1/40) are due to the intensity increase of the guanine, uracil, and phosphate bands upon SFN complexation and approves major interaction of sulforaphane with guanine N7, uracil O2 and backbone PO_2_ group.

Additional evidence regarding sulforaphane–RNA interaction comes from the UV results. An increase in the intensity of RNA band at 258 nm can be related to the interaction of sulforaphane with RNA (Fig 4B). Similar spectral changes were observed in other RNA adducts [43].

On the other hand, the UV band at 245 nm characteristics of sulforaphane absorption showed major red shift and appeared at 258 nm upon RNA interaction. The observed spectral changes can be indicative of structural alterations of the SFN upon its complexation with RNA (Fig 4B).

### DNA and RNA conformation

No alterations of B-DNA structure was observed upon SFN-DNA complexation as a result of no major spectral changes for B-DNA marker bands at 1226 cm^-1^ (PO_2_ stretch), 1714 cm^-1^ (mainly guanine), and 836 cm^-1^ (phosphodiester mode) (Fig 3A). In a B to A transition, the marker band at 836 cm^- 1^ shifts toward a lower frequency at about 825–800 cm^-1^, the guanine band at 1714 cm^-1^ appears at 1700–1695 cm^-1^, and the phosphate band at 1226 cm^-1^ shifts toward a higher frequency at 1240–1235 cm^- 1^ [45–48]. In a B to Z conformational change, the sugar–phosphate band at 836 cm^-1^ appears at 800–780 cm^-1^, the guanine band displaces to 1690 cm^-1^, and the phosphate band shifts to 1216 cm^-1^ [47, 48]. In the SFN-DNA complex (r = 1/5), shifting of the B-DNA marker bands at 1714 to 1700 cm^-1^, at 833 to 826 cm^-1^ and at 1226 to1230 cm^-1^ is indicative of DNA conformational change from B to A upon sulforaphane interaction (Figs 3A and 2A).

In the sulforaphane–RNA complexes, RNA remains in A-conformation. The lack of major shifting of A-RNA marker bands at 1700–1688 (guanine), 1240–1247 (phosphate), 861–867 (ribosephosphate), and 815–809 cm^-1^ (phosphodiester) is indicative of RNA remaining in A-conformation upon sulforaphane complexation (Figs 3B and 2B). [44, 49–51].

### Stability of sulforaphane–DNA and sulforaphane–RNA complexes

The sulforaphane–DNA and—RNA binding constants were determined as described in the experimental section (UV–visible spectroscopy). The UV absorption spectra of sulforaphane–DNA and—RNA complexes are shown in Fig 4A and 4B. The calculations of the overall binding constants were carried out using UV spectroscopy as previously reported [30]. Concentrations of the complexed ligand were determined by subtracting absorbance of the free DNA at 259 nm and RNA at 258 nm from those of the complexed. Concentration of the free ligand was determined by subtraction of complexed ligand from total ligand used in the experiment. Our data of 1/[ligand complexed] almost proportionally increased as a function of 1/[free ligand] (Fig 4). The double reciprocal plot of 1/(A-A_0_) versus 1/(sulforaphane concentration) is linear, and the binding constant (K) can be estimated from the ratio of the intercept to the slope (Fig 4), where A_0_ is the initial absorbance of the free DNA at 259 nm and free RNA at 258 nm, and A is the recorded absorbance of DNA and RNA in the presence of different sulforaphane concentrations. The overall binding constants are estimated to be K_(SFN–DNA)_ = 3.01 (± 0.035) ×10^4^ M^-1^ and K_(SFN–RNA)_ = 6.63 (±0.042) ×10^3^ M^-1^. The affinity of sulforaphane–DNA and—RNA is in the order of SFN–DNA>SFN–RNA. Sulforaphane binding to DNA is stronger, since DNA is double helix, but RNA is not. These binding constants are consistent with FTIR results, which we concluded a stronger interaction of sulforaphane with DNA than that of RNA complexes.

### Docking study

**Fig 5:**
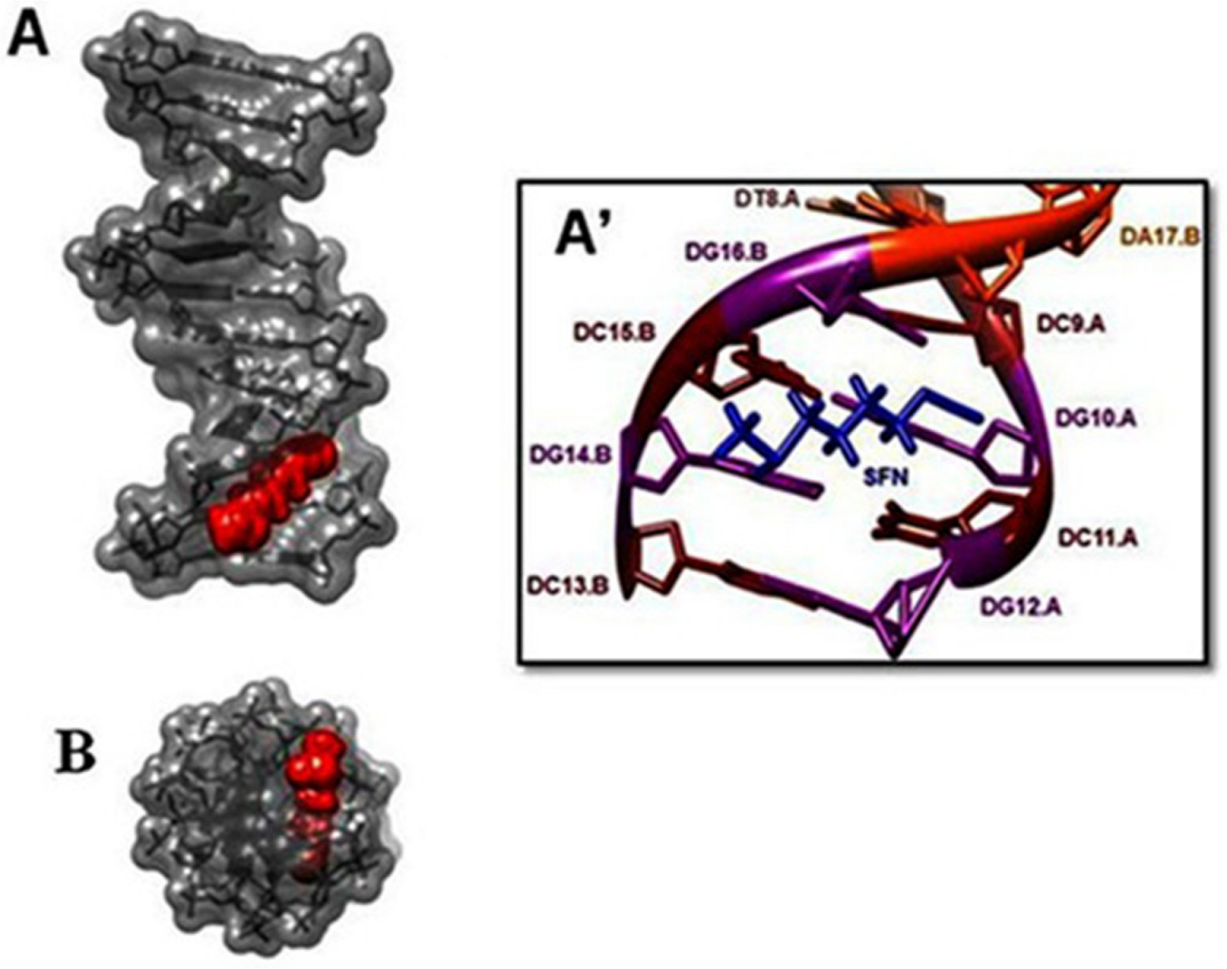
Docking structure between d(CGCGAATTCGCG) (PDB 1BNA-DNA) and SFN. (A) Surface representation of d(CGCGAATTCGCG) complexes with SFN (Display side). (A') Close up view of d(CGCGAATTCGCG) complexes with SFN. (B) Surface representation of d(CGCGAATTCGCG) complexes with SFN (Display top).

**Fig 6:**
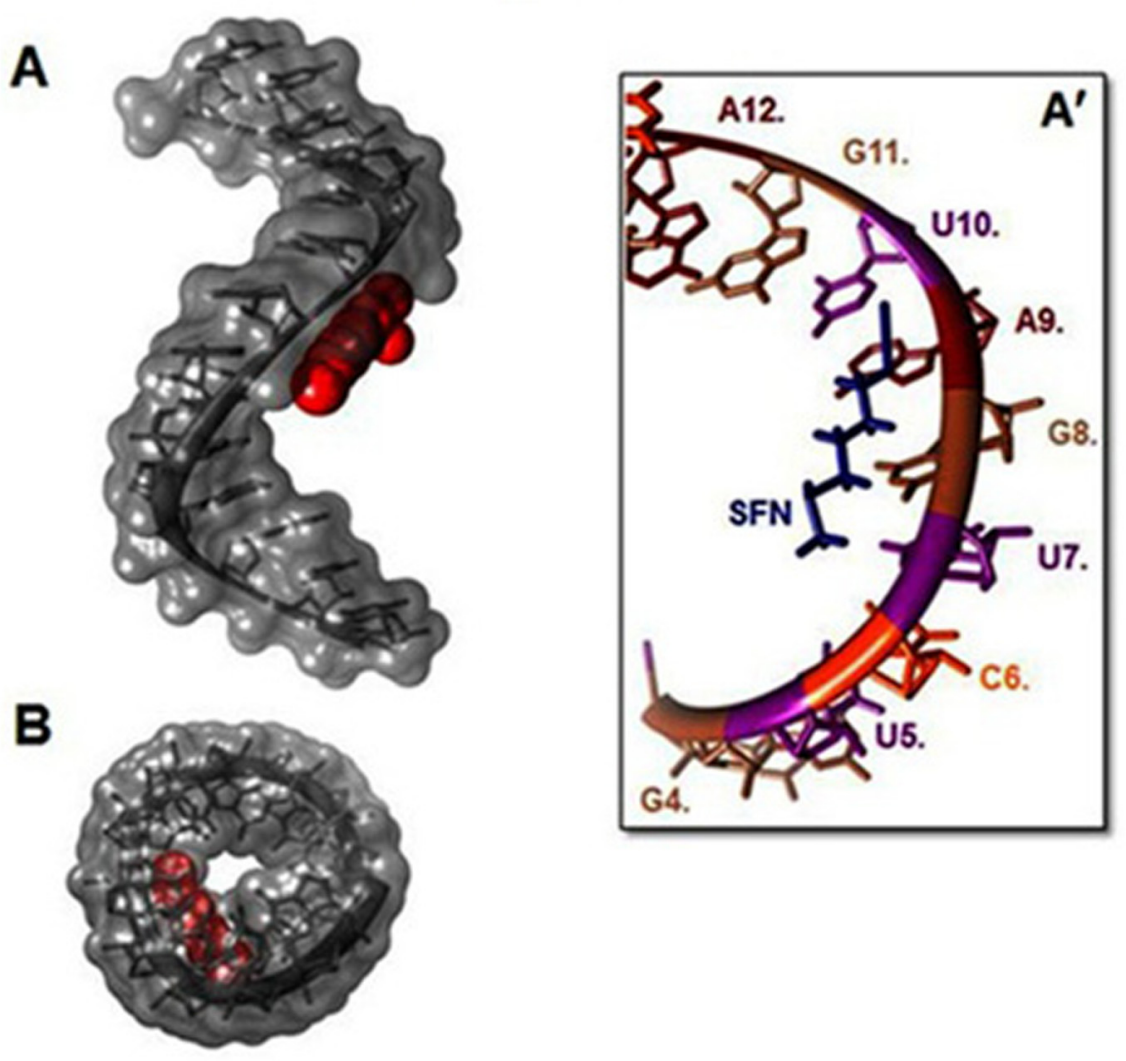
Docking structure between (PDB 3CZW) and SFN. (A) Surface representation of (PDB 3CZW) complexes with SFN (Display side). (A') Close up view of (PDB 3CZW) complexes with SFN. (B) Surface representation of (PDB 3CZW) complexes with SFN (Display top).

**Fig 7:**
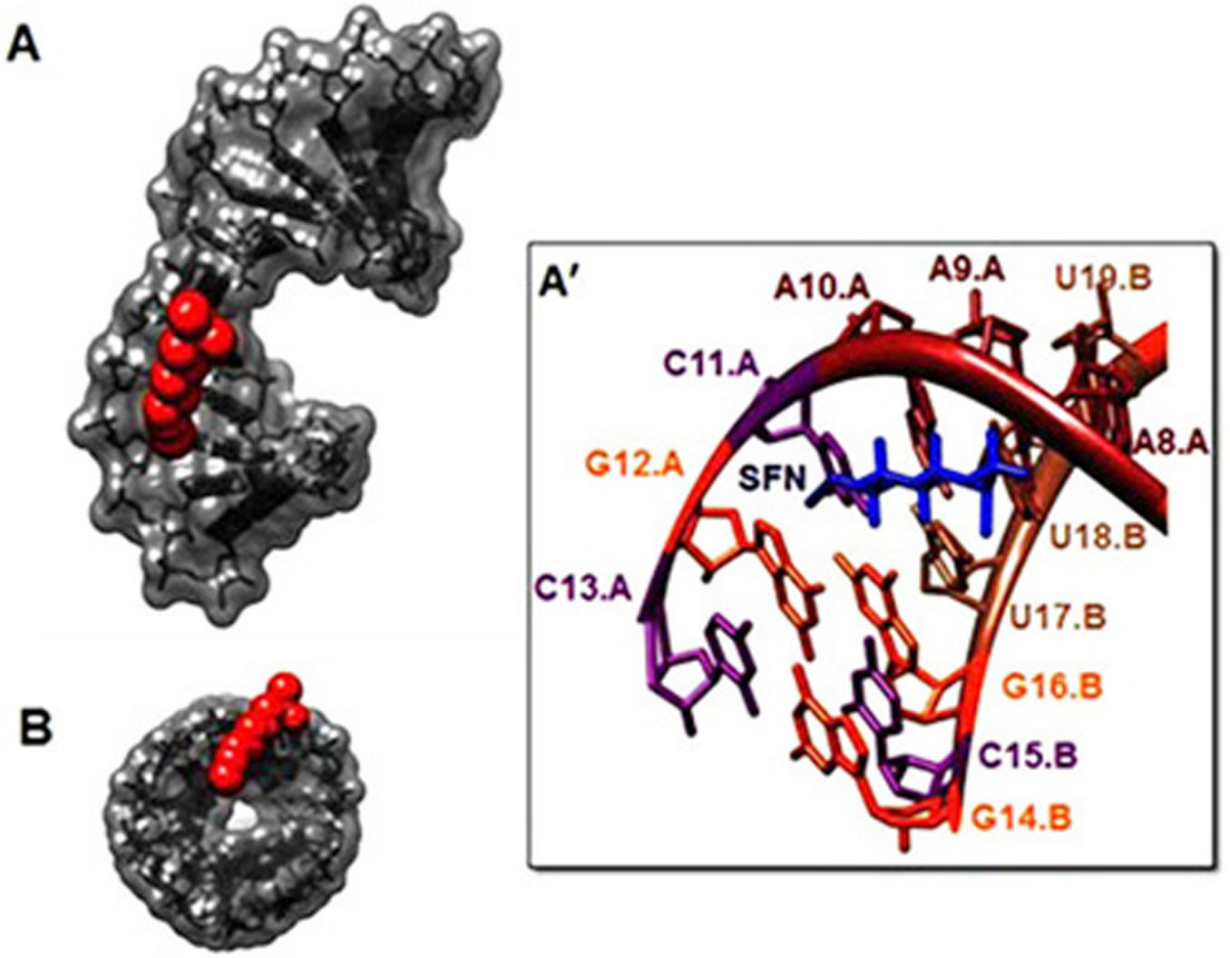
Docking structure between (PDB 2R22) and SFN. (A) Surface representation of (PDB 2R22) complexes with SFN (Display side). (A') Close up view of (PDB 2R22) complexes with SFN. (B) Surface representation of (PDB 2R22) complexes with SFN (Display top).

Our results from FTIR and UV-Visible spectroscopy are accompanied by docking experiments. In order to determine the preferred binding sites on DNA and RNA, the sulforaphane were docked to DNA and RNA. The dockings results are shown in Figs 5, 6 and 7.

For docking, PDB 3CZW was chosen as single stranded RNA candidate and PDB 2R22 as double stranded RNA candidate. PDB 3CZW and PDB 2R22 include all kinds of bases (A,U,G and C). PDB 1BNA-DNA with minor and major grooves including all kinds of bases (A,T,G and C) was chosen as candidate for double stranded DNA.

The models show that SFN is surrounded by C13.B, G14.B, C15.B, G16.B, C9.A, G10.A, C11.A, G12.A (Fig 5A′) and phosphate groups with a binding energy of -4.499 kcal/mol for DNA. SFN was surrounded by U7, G8, A9, U10 (Fig 6A′) and phosphate groups with a binding energy of -4.41 kcal/mol for single strand RNA. SFN was surrounded by C11.A, A10.A, A9.A, A8.A, G16.B, U17.B, U18.B (Fig 7A′) and phosphate groups with a binding energy of -4.05 kcal/mol for double strand RNA. In several data derived from SFN and 1BNA-DNA, 2R22 and 3CZW the docking shows different mood energy intercalations, the structure was selected which has the most compatibility with FTIR results. Spectroscopic evidence, FTIR and UV results showed both intercalation and external binding of SFN to DNA and RNA. The selected docking data showed perpendicular intercalation into oligonucleotides (1BNA-DNA, 2R22 and 3CZW) (Figs 5A′,6A′ and 7A′).

## Conclusion

Our study provided important quantitative data on the binding affinity of sulforaphane to DNA and RNA. Direct binding experiments and DNA denaturation assays need to be done to determine the effect of low and high concentrations of SFN on polynucleotides structure.

We also showed distinct differences in sulforaphane binding to these biopolymers. Interaction of sulforaphane with DNA and RNA can be used to gain insight into the mechanism of action of sulforaphane in cancer therapy. Based on our spectroscopic results and docking studies, the following points are important; sulforaphane binds DNA and RNA *via* both intercalation and groove binding with the order of stability SFN-DNA>SFN-RNA. At high SFN concentrations, DNA conformational changed from B to A occurred, while RNA remained in A-family structure.
